# Weight Reduction by the Low-Insulin-Method—A Randomized Controlled Trial

**DOI:** 10.3390/nu12103004

**Published:** 2020-09-30

**Authors:** Martin Röhling, Katharina Martin, Sabine Ellinger, Michael Schreiber, Stephan Martin, Kerstin Kempf

**Affiliations:** 1 West-German Centre of Diabetes and Health, Dusseldorf Catholic Hospital Group, 40591 Dusseldorf, Germany; stephan.martin@vkkd-kliniken.de (S.M.); kerstin.kempf@wdgz.de (K.K.); 2 Hochschule Niederrhein, University of Applied Sciences, 41065 Mönchengladbach, Germany; katharina.martin@ish.de (K.M.); sabine.ellinger@hs-niederrhein.de (S.E.); 3 Psychiatric-Psychotherapeutic Day-Care Hospital, Dusseldorf Catholic Hospital Group, 40591 Dusseldorf, Germany; michael.schreiber@vkkd-kliniken.de; 4 Faculty of Medicine, Heinrich Heine University Dusseldorf, 40225 Dusseldorf, Germany

**Keywords:** weight loss, telemedical coaching, meal replacement therapy, RCT

## Abstract

Continuous high insulin levels are associated with weight gain and lead to cardiometabolic diseases. Therefore, we have developed the Low-Insulin-Method and integrated it into the multi-component, occupational healthcare program SHAPE-AND-MOTION-Medical-Accompanied-Slimming (SAMMAS) to reduce daily insulin levels for long-term weight reduction in overweight or obesity. Employees were randomized into a starting intervention group (SI, *n* = 15) or waiting list control group (WL, *n* = 15). SAMMAS consisted of group-based seminars, low-carbohydrate nutrition including formula diet, continuous glucose monitoring, telemetric monitoring, and telemedical coaching. Both groups received telemetric devices at baseline. Intention-to-treat analyses were performed after 12, 26, and 52 weeks. The estimated treatment difference in weight reduction after 12 weeks, which is the primary endpoint of the study, showed a pronounced effect in favour of SI (−6.3 kg with (95% confidence interval) (−7.4; −4.5) (*p* < 0.001)) after 12 weeks. Furthermore, SI improved fasting blood glucose, HbA1c, quality of life, fasting insulin, blood pressure, and eating behaviour (all *p* < 0.05) in the within-group analysis, while WL did not. After 26 and 52 weeks, weight reduction could be maintained in the whole group (both groups together) by −6.7 kg (−9.5; −3.8) (*p* < 0.001) and −6.1 kg (−9.2; −2.7) (*p* < 0.01). SAMMAS supports clinically relevant weight reduction and long-term weight loss maintenance in individuals with overweight or obesity.

## 1. Introduction

In industrialized countries with a Western diet, three, mostly carbohydrate-rich meals have been established, with an additional evening snack or several snacks over the course of the day [1]. Such excessive, carbohydrate-rich nutrition in combination with low physical activity (as part of a poor lifestyle) supports the development of overweight, obesity and cardiometabolic diseases like type 2 diabetes mellitus [2]. Starch-free and complex carbohydrates (e.g., fibre from whole grain) are associated with a longer life expectancy [3] and reduced blood glucose levels [4]. However, the consumption of less complex carbohydrates leads to an insulin secretion, which not only mediates the uptake of glucose from the blood into the cells, but, already at a lower concentration by a factor of 6, stops lipolysis for several hours [5]. The duration of the blockade depends, among other things, particularly on the BMI. Compared to lean individuals, people with overweight or obesity have increased fasting insulin levels, show sharp rises in insulin after carbohydrate consumption, and their postprandial insulin levels remain elevated for longer [6]. Moreover, hyperinsulinemia, not only caused by consistent and excessive carbohydrate consumption, but maybe also by food additives like emulsifiers, and probably by sedentary lifestyle and other environmental factors, is thought to be one of the main causes of the increasing prevalence of overweight and obesity [7,8,9,10]. Accordingly, lifestyle interventions, comprising healthy diet and physical activity, are recommended for individuals with overweight or obesity [11,12,13]. Despite immense initiatives all over the world to stop obesity [14], the incidence and prevalence of overweight and obesity has increased dramatically [2]. To counter this, in recent years, various diet approaches have become popular [15,16,17,18,19]. However, these different nutritional approaches primarily focus on the composition of food and often disregard the physiological reactions in the body.

Therefore, we have developed the Low-Insulin-Method and integrated it into to multicomponent occupational healthcare program SHAPE-AND-MOTION-Medical-Accompanied- Slimming (SAMMAS) that aims to lower insulin levels in order to allow for effective lipolysis. In previous studies we have shown that lifestyle interventions of our Low-Insulin-Method, including a low-carbohydrate meal replacement [20,21,22], self-monitoring of blood glucose (SMBG) [23,24], telemedical monitoring with telemetric devices [25,26] and telemedical coaching including medical mental motivation [20,27], lead to clinically relevant improvements in body weight and other cardiometabolic parameters in different cohorts. In this randomized controlled trial (RCT), we combined these evaluated lifestyle intervention components and tested the hypotheses that (i) the application of SAMMAS in an occupational health care setting for 12 weeks supports clinically relevant weight loss in individuals with overweight or obesity compared to a control group and (ii) the long-term effects on weight-loss as well as on laboratory, anthropometric and behavioural parameters after 52 weeks.

## 2. Materials and Methods

### 2.1. Study Population

As part of an occupational healthcare initiative, employees with overweight or obesity in the Düsseldorf Catholic Hospital Group in Düsseldorf, Germany, were offered the chance to participate in the present study. Eligible volunteers (inclusion criteria: age ≥18 years, body mass index (BMI) ≥25 kg/m^2^; exclusion criteria: acute diseases, chronic diseases such as cancer, chronic obstructive pulmonary disease, asthma, dementia, chronic bowel disease, psychosis, liver cirrhosis, nephropathy, renal insufficiency with glomerular filtration rate (GFR) < 30mL/min/1.73 m^2^ (but not type 2 diabetes), severe illness with in-patient treatment during the last 3 months, weight change >2 kg/week during the last month, smoking secession during the last 3 months, intake of drugs for active weight reduction, pregnancy, breastfeeding) were randomized according to an electronically generated randomization list into two parallel groups. In detail, each participant was assigned a serial study identifier (ID). The ID of each participant was stored in a closed envelope with the group assignment. The first participant was enrolled on 11 January 2018 and the last participant finished the study on 10 April 2019. The study was conducted at the West-German Centre of Diabetes and Health (WDGZ) in Düsseldorf, Germany, in accordance with the ethical standards laid down in the 1964 Declaration of Helsinki and its later amendments. The research protocol was approved by the ethics committee of the Ärztekammer Nordrhein, Düsseldorf (No. 2012212) as well as registered at clinicaltrials.gov (trial-ID: NCT03417674), and the study adheres to CONSORT guidelines. All participants gave written informed consent prior to their inclusion in the study.

### 2.2. Study Design

**Figure 1:**
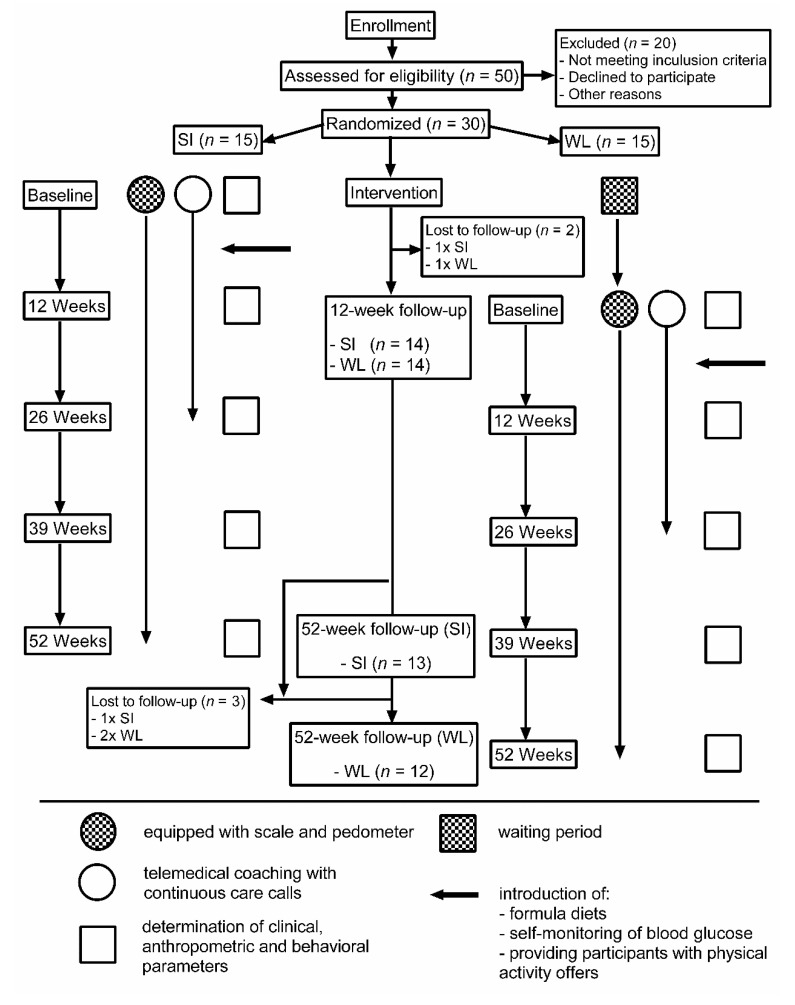
Flow diagram. SI, starting intervention group; WL, waiting list control group.

Eligible participants were randomly assigned into either a starting intervention group (SI) or a waiting list control group (WL). After baseline examination, all participants were equipped with telemetric devices (scales and pedometers). The SI-group started immediately with the SAMMAS intervention with weekly contacts during the 12-week intervention phase. The contacts consisted of a group-based education program (seven theoretical sessions and two practical modules lasting 90 min each) and telemedical coaching (four care calls lasting 20–30 min each). At the same time, the WL-group continued their habitual lifestyle and received no further information. During the follow-up phase, SI-participants received monthly care calls. In parallel (when the SI-group finished the 12-weeks phase), the WL-group started with the 12-weeks intervention phase and underwent the same treatment as the SI-group. The participants, the study nurse, and the outcome assessor were blinded for sequence of allocation concealment. The data analyst was blinded after assignment to the intervention. A detailed summary of the study design and interventions can be seen in Figure 1.

### 2.3. The SAMMAS Components

*Telemetric devices*. Both groups were equipped with telemetric scales (smartLAB scale W; HMM Holding AG, Dossenheim, Germany) and pedometers (smartLAB walk P+; HMM Holding AG, Dossenheim, Germany) at baseline, automatically transferring recorded data into a personalized online portal. These data could be monitored by both participants and the lifestyle coaches of the WDGZ. Participants were instructed to measure their weight and steps continuously throughout the whole study period.

*Education program*. The seminar-based education program consisted of seven theoretical (i–vii) and two practical modules (viii–ix) ((viii) exercise and (ix) cooking) addressing different lifestyle-related topics (90 min per seminar session). The main theoretical topics were: (i) low-carbohydrate and healthy diet (e.g., foods with complex carbohydrates or rich in fibre, as well as sources of simple sugar), (ii) insulin–blood glucose–lipolysis relationship, (iii) meal replacement therapy by formula diets (e.g., possible diet regimes), (iv) SMBG by continuous-glucose-monitoring systems (e.g., influence of different food ingredients on the glucose level), (v) eating behaviour (e.g., control of hunger or suggestibility), (vi) physical activity (e.g., effect of different types of physical activity on energy consumption), and (vii) acid-base balance (e.g., keto-acetonic effect of limiting carbohydrate consuming) (seminar order: i–iv, ix, v–vi, viii, vii). The program was developed and presented by academic experts, such as nutritionists, exercise scientists, biologists and physicians in cooperation with psychologists.

*Low-carbohydrate nutrition and meal replacement therapy.* The study participants were provided with information about the current scientific results of meal replacement with formula diets in overweight/obesity management as well as general facts about the impact of low-carbohydrate meals on blood glucose and insulin levels. As part of the nutritional intervention, participants could freely choose between self-assembled low-carbohydrate nutrition or meal replacement with a formula diet. In each case, the nutrition should consist of no more than 30% carbohydrates [28]. If participants used the low-carbohydrate nutritional approach, they had to reduce their total amount of carbohydrates and primarily consume starch-free and complex carbohydrates (especially fibre from whole grain) and abstain from less complex carbohydrates (e.g., sweets, pasta, bread). In contrast, participants were advised to increase intake of protein-rich components and unsaturated fatty acids (e.g., olive oil, fish).

Participants who decided to follow a systematic meal-replacement could freely chose a low-carbohydrate formula diet consisting of ~30% carbohydrates and ~50% protein (e.g., *Almased*^®^, Almased Wellness GmbH, Germany, *Madena Pro Classic*^®^, MADENA GmbH & Co. KG, Cologne, Germany or others) and to replace all three main-meals as described previously [20]. In brief, participants replaced three of three meals during the first week. No additional snacks were permitted. During weeks 2–4, these participants should re-introduce a low-carbohydrate lunch; furthermore, two of three meals were replaced by formula diet. With the beginning of week 5, an additional low-carbohydrate breakfast should be re-introduced while one of three meals still had to be replaced by formula diet. The re-introduction phase was accompanied by continuous glucose monitoring (and was also provided to the participants who decided to abstain from meal replacement therapy), in order to learn more about the impact of individual foods on glucose levels. The low-carbohydrate diet had to be continued until the end of the study after 52 weeks. Moreover, participants were instructed to replace meals whenever necessary if weight gain took place during the study. In order to improve study compliance, consumed meals should be documented by taking pictures, but this information was not statistically investigated.

To evaluate diet adherence or meal replacement regimen compliance, participants were advised to record their meals in a diet diary and to focus on a low-carbohydrate nutrition. Furthermore, all participants were supervised by coaches during the telemedical coachings and were asked to provide information on their nutritional behaviour during the study and whether and how often they replaced their meals with formula diets.

*Continuous glucose monitoring*. Participants were equipped with a continuous glucose monitoring (CGM) system (FreeStyle Libre^®^, Abbott Diabetes Care, Alameda, SK, Canada) for two weeks during weeks 3 and 4 and instructed to document their lunch and the corresponding glucose values before and 1.5–2.0 h after each meal. Additionally, they should try out the effects of intensive exercise on blood glucose levels. Participants were encouraged to adapt their meals and habits, aiming to keep glucose levels within a normal range. In case of nutrition-related uncertainties, they received help from the coaches. Anti-diabetic medication (i.e., Metformin) was monitored and adjusted by treating physicians. The CGM system primarily served as a pedagogic tool to visualize the influence of foods on the glucose level to the participants and to support self-empowerment.

*Telemedical coaching*. The education program was continuously supported by telemedically supported coaching [25]. Coaching sessions included information about coping strategies for lifestyle changes, healthy diet for maintaining insulin levels low, physical activity, motivation and overweight or obesity-related diseases like type 2 diabetes. Data, which had been acquired from the telemetric devices (which could be monitored by coaches and participants during the whole study), were discussed within the coaching sessions (i.e., steps, weight). Furthermore, participants were motivated to achieve their individually determined goals (i.e., weight goals, step counts) using the medical mental motivation program [25,27], and negative developments regarding recorded data were evaluated and discussed with the participants. Therefore, coaching sessions were also used to increase the study compliance and adherence of probands by coaches. Moreover, participants were asked for the frequency and amount of formula diet used, as well as the composition of meals, to increase compliance to the low-carbohydrate diet. Deviations from the systematic formula diet regimen or low-carbohydrate diet approach were discussed and participants were advised to follow the guidelines of the intervention program. The frequency of coaching sessions comprised four appointments during the first 12 weeks of intervention, and three calls during the follow-up period from week 13 to 26. The telemedical coaching calls were performed by specially trained lifestyle coaches and each call lasted for 20–30 min.

*Physical activity*. The program also provided guidance on how to change physical activity habits and how to react to elevated blood glucose levels. Based on current guidelines [13], participants were advised to increase physical activity and advised on how to perform it properly. These theoretical recommendations were underpinned by practical exercise sessions in cooperating gyms and sports clubs during the 12-week intervention phase.

### 2.4. Measurements

Participants visited the study centre after an 10 h overnight fast at baseline and after 12, 26, and 52 weeks of intervention for the determination of anthropometric and clinical data (i.e., age, sex, body weight, height, body mass index (BMI), waist circumference, fat mass (FM), systolic blood pressure (SBP), diastolic blood pressure (DBP), total cholesterol, high-density lipoprotein (HDL)-cholesterol, low-density lipoprotein (LDL)-cholesterol, triglycerides, fasting blood glucose, fasting insulin, homeostasis model assessment of insulin resistance (HOMA-IR) and haemoglobin A1c (HbA1c). Body weight was measured in light clothing and FM was predicted (based on the four-compartment model [29]) using a body composition scale (Seca mBCA515, Seca, Hamburg, Germany) which was validated for use to determine FM (predictive formulas can be seen in the reference used) [30]. Blood pressure was measured on both arms after a five-minute rest in a sitting position. Venous blood was collected by inserting an intravenous cannula into the forearm vein and laboratory parameters were analysed at a local laboratory as described previously [20]. Validated self-reporting questionnaires were used to assess quality of life (Short Form (SF) 12 Questionnaire) [31], physical activity (Freiburger Questionnaire for Physical Activity (FFkA)) [32] and eating behaviour (German version of the ‘Three-factor Eating Questionnaire’ (TFEQ)) [33] and cardiovascular disease risk (Framingham risk score (FRS)) [34].

### 2.5. Statistical Analysis

Data are presented as means and standard deviations (mean ± SD), means and 95% confidence intervals (mean (95% CI)), or percentages, as appropriate. Per-protocol (PP) and intention-to-treat (ITT) analyses were performed. Missing values were imputed by the ‘last observation carried forward (LOCF)’ approach. Primary endpoint was the difference in weight reduction after 12 weeks between both groups, determined by the estimated treatment difference (ETD). Previous own data have indicated that, with the use of a low-carbohydrate meal replacement, a reduction in body weight of 6.9 ± 5.0 kg can be achieved, while a reduction of 1.0 ± 2.0 kg was assumed for the control group equipped with telemetric devices [20,21]. Furthermore, a study previously published by us showed a significant weight reduction of 3.1 ± 4.8 kg in overweight employees after 12 weeks of intervention with telemedical coaching and telemetric devices [25]. We, therefore, estimated a summative effect of 7.5 ± 6.0 kg in the present study. In order to measure differences between both intervention groups with a power of 90% and a level of significance of 5%, a sample size calculation revealed that at least *n* = 12 datasets would be needed. Since a dropout rate of about 20% was assumed, the plan was to recruit a total of 15 participants per group.

Secondary outcomes comprised differences in BMI, cardiometabolic risk factors, eating behaviour, quality of life, and physical activity after 12 weeks of intervention between both groups. Multivariable regression analyses were carried out to investigate group differences and were adjusted for potential confounders such as age, sex, BMI, baseline values and multiple testing. Tertiary endpoints focused on changes in weight, BMI, HbA1c and fasting insulin from baseline to week 12, 26 and week 52 within the whole group. These were analysed using mixed models adjusting for repeated measurements, baseline values and multiple testing. In order to analyse the predictive value of insulin on weight reduction, the participants were stratified in tertiles according to their fasting insulin levels at baseline to investigate the weight change from baseline to week 12, 26 and 52. Furthermore, a bivariate correlation analysis was performed to investigate the association between weight change and the change in fasting insulin following the intervention. A further exploratory analysis investigated the differences in changes in the WL-group, comparing the initial waiting period with the first 12 weeks of intervention.

Non-parametric data were analysed with Mann–Whitney U, Wilcoxon, and Friedman test and parametric data with Student’s *t*-test, paired *t*-test, and analysis of variance with repeated measures to determine differences between groups following the intervention. Dichotomous variables as well as frequencies were compared by Fishers exact test, McNemar test or Cochrane Q test. All statistical tests were two-sided, and the level of significance was set at α = 0.05. *p*-values were adjusted for multiple comparisons using Bonferroni correction. All analyses were performed using SPSS 22.0 (SPSS Inc., Chicago, IL, USA) and GraphPad Prism 6.04 (GraphPad Software, San Diego, CA, USA).

## 3. Results

Out of 50 subjects who were interested in our study, 36 met criteria for eligibility. However, six of them declined to participate or were excluded for other reasons. Finally, thirty participants were included in the study and randomized into either the SI-group (*n* = 15), or the WL-group (*n* = 15). The demographical and clinical characteristics of both groups did not differ at baseline (Table 1). Fourteen (93%) participants of the SI-group and 14 (93%) of the WL-group completed the 12-week intervention period. Follow-up data obtained 52 weeks after the beginning of the study were available from 25 participants (80%). The main reasons for dropouts were ‘personal/private reasons’ (*n* = 3), change of employer (*n* = 1) and lack of time (*n* = 1). Participants who completed the intervention and follow-up phase and those who dropped out or were lost to follow-up did not differ significantly. No adverse effects have been reported.

### 3.1. Results Report of SI and WL (First 12 Weeks)

*Primary endpoint*. Participants of the SI-group significantly reduced weight compared to the WL-group after 12 weeks of intervention in the PP and ITT analysis (*p* < 0.001) (Table 2). This reduction remained statistically significant after Bonferroni correction and adjustment for age, sex, BMI and baseline values in both analyses (*p* ≤ 0.001). The ETD in weight reduction after 12 weeks was −6.3 kg with 95% CI (−7.40, −4.50; *p* < 0.001) (−6.8% (−8.0, −3.9; *p* < 0.001)) in PP analysis and −5.9 kg (−8.10 kg, −4.10; *p* < 0.001) (−6.3% (−7.8, −3.7; *p* < 0.001)) in the ITT analysis.

*Secondary endpoints*. Compared to the WL-group, SI-group significantly improved in BMI, waist circumference, fat mass, and all variables of eating behaviour after 12 weeks of intervention in both PP and ITT analysis (all *p* < 0.05). After adjusting for age, sex, BMI, baseline values, and multiple testing, improvements in the SI-group in BMI, waist circumference, fat mass and eating behaviour (*hunger*) were still significantly different from the WL-group (*p* ≤ 0.001). The SAMMAS intervention also led to improvements in the WL-group when investigating the differences in changes of weight, BMI, waist circumference, and fat mass (all *p* < 0.05 (PP and ITT)) comparing the initial waiting period with the first 12 intervention weeks (Supplementary Table S1).

### 3.2. Results Report of the Total Sample (Until 52 Weeks)

**Figure 2:**
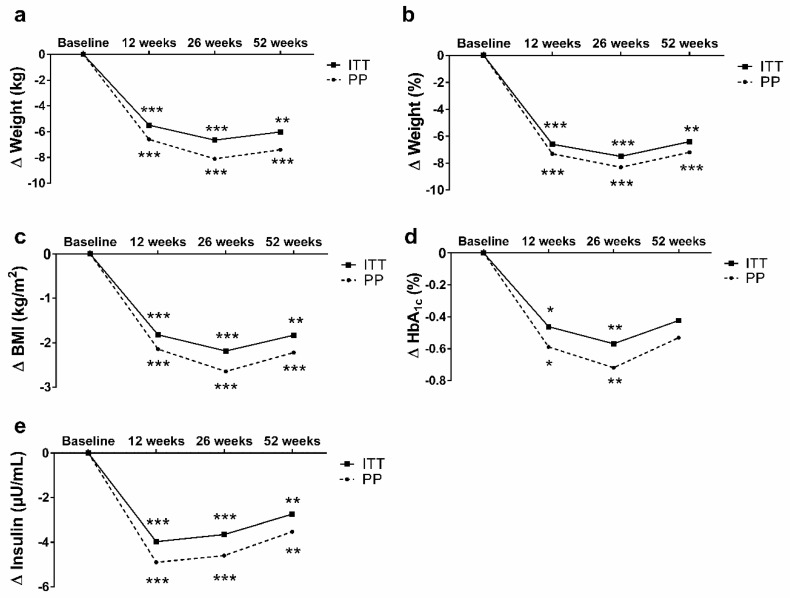
Mean changes in weight (**a**,**b**), BMI (**c**), HbA1c (**d**) and fasting insulin (**e**) after 12, 26, and 52 weeks of *n* = 30 (ITT) or *n* = 25 (PP). Within-group changes were analysed using mixed models adjusting for repeated measurements, baseline values and multiple testing. *** *p* < 0.001 vs. baseline; ** *p* < 0.01 vs. baseline; * *p* < 0.05 vs. baseline; BMI, body mass index; ITT, intention-to-treat analysis; HbA1c, haemoglobin A1c; PP, per-protocol analysis.

*Tertiary endpoints*. Changes in weight (absolute and relative), BMI, HbA1c and fasting insulin within the whole group after 12, 26 and 52 weeks of intervention are shown in Figure 2a–e. Improvements in weight (26 weeks: ITT −6.7 kg (95% CI: −9.5; −3.8) (*p* < 0.001) and PP −9.1 kg (95% CI: −13.2; −5.1) (*p* < 0.001); 52 weeks: ITT −6.1 kg (95% CI: −9.2; −2.7) (*p* < 0.01) and PP −7.4 kg (95% CI: −11.4; −3.4) (*p* < 0.01)) as well as BMI (26 weeks: ITT −2.2 kg/m² (95% CI: −3.2; −1.2) (*p* < 0.001) and PP −3.0 kg/m² (95% CI: −4.4; −1.7) (*p* < 0.001); 52 weeks: −1.8 kg/m² (95% CI: −3.0; −0.7) (*p* < 0.01) and PP −2.2 kg/m² (95% CI: −3.7; −0.8) (*p* < 0.01)) were observed in the whole group after 12, 26, and 52 weeks of follow-up. These differences were still significant after Bonferroni correction.

**Figure 3:**
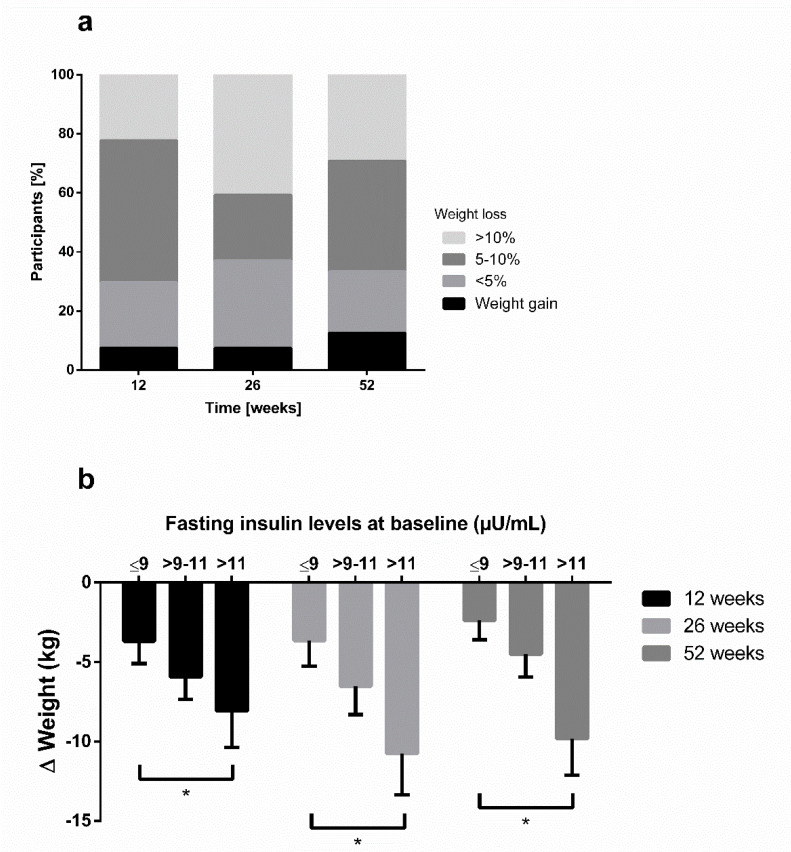
Changes in body weight of the total sample. PP-analysis with *n* = 25. (**a**) Frequency of categories of weight changes throughout the study were compared by using χ2 test (* *p* < 0.05). (**b**) Tertile stratification in baseline fasting insulin levels for weight changes in all three groups after 12, 26, and 52 weeks. Multivariable regression analyses were performed to investigate group differences and were adjusted for baseline values and multiple testing. * *p* < 0.05.

After 12 weeks, 70% of participants were successful in losing at least 5% of body weight and 70% maintained this 5% weight loss until 52 weeks of follow up. Of *n* = 20 participants with a BMI ≤35 kg/m², 65% accomplished a weight reduction of >5% after 12 weeks of intervention, and 60% maintained it until week 52. A total of 20% (75%) of participants with a BMI > 35 kg/m² at baseline (*n* = 10) achieved a weight loss of >10% (>5%) of body weight after 12 weeks and the percentage increased to 30% (66%) after 52 weeks. The distribution of categories for weight change after 12, 26 and 52 weeks is displayed in Figure 3a (PP).

Changes in glucometabolic parameters (HbA1c and fasting insulin) were already significantly different after 12 and 26 weeks of intervention. However, only fasting insulin was still significantly different after 52 weeks of follow-up (ITT: −2.8 µU/mL (95% CI: −4.4; −1.1) (*p* < 0.01) and PP −3.5 µU/mL (95% CI: −5.5; −1.5] (*p* < 0.01)) and Bonferroni correction. The Framingham Risk Score significantly decreased after 12 (−1.7 points, *p* < 0.01), 26 (−2.0 points, *p* < 0.001) and 52 weeks (−2.0 points, *p* < 0.001) (PP).

In order to analyze the effect of hyperinsulinaemia on weight loss, we stratified the participants into three categories according to their fasting insulin levels at baseline. This post-hoc analysis showed higher weight losses through low-carbohydrate meal replacement in participants with elevated fasting insulin levels compared to those with lesser insulin levels (*p* < 0.05) (Figure 3b). Furthermore, a bivariate correlation analysis showed a significant association of Δ body weight and Δ fasting insulin (r = 0.481, *p* = 0.017) after 52 weeks.

## 4. Discussion

In the present study, we could demonstrate that the Low-Insulin-Method included in the multi-component, occupational healthcare SAMMAS program focusing on hypocaloric, low-carbohydrate diet, meal replacement therapy, continuous glucose monitoring, telemonitoring and telemedical coaching, and aiming to lower insulin levels in order to allow effective lipolysis, leads to a significant weight reduction in individuals with overweight or obesity. This weight reduction was clinically relevant after 12 weeks of intervention, and could be sustained until 52 weeks of follow-up. Furthermore, 70% of all participants could maintain a long-term weight reduction of at least 5%.

The Finnish Diabetes Prevention Study (DPS) [35], the Diabetes Prevention Program (DPP) [36] and other large lifestyle intervention studies [37] achieved mean weight losses after one year of −4.5, −6.5, and −2.5 kg, respectively. However, mean weight loss in the present study is considerably higher (PP-analysis: −7.4 kg) after 52 weeks of intervention with a primary non-diabetic cohort. Moreover, the extent of weight loss is in line with other studies using very low-calorie (<800 kcal per day) or low-energy liquid-formula diet (>800 kcal per day), with mean body weight reduction ranging from 8.9 to 15.0 kg in obese subjects (BMI: 35.5–42.6 kg/m^2^) [20,38], although a low-calorie approach starting with ~1200 kcal/day was used in just the first week.

Our education program focused on the main topic of carbohydrate-induced hyperinsulinemia, which seems to be one of the main driving forces for the development of obesity and diabetes [7]. Therefore, the theoretical sessions focused on the insulin–blood glucose relationship and their intercorrelations with today’s diet, especially regarding carbohydrates. Our low-carbohydrate meal replacement approach is based on the recommendations of the current guidelines of the American Diabetes Association (ADA) [13] and the consensus reports of both ADA and the European Association for the Study of Diabetes (EASD) [11,12]. Furthermore, studies have shown that a reduced carbohydrate intake [17] and a reduced number of carbohydrate-containing meals [39] trigger fast effects in glucose metabolism, especially in terms of body weight, insulin resistance/sensitivity, and beta cell function [40].

The behaviour training of our study is mainly based on the regularly performed telemedical coachings. In previous studies, we could already show that telemedical coaching in combination with telemetric devices contributes to weight loss in overweight patients with and without diabetes [20,25]. Our results are in line with Appel et al., who demonstrated that telemedical coaching, in comparison to in-person support, contributes to a similar extent of weight reductions of more than 5% during a weight-loss intervention program [41]. These findings emphasize the potential of telemedical coaching in combination with telemonitoring and indicate an effective approach for weight management. However, only telephone-call-based interventions without telemedical coaching were not sufficient to sustainably change behaviour and reduce weight, and demonstrated higher dropout rates as well [42]. This relation elucidates the necessity of external experts and coaching in telemedical interventions.

The reduction in body weight in the present study was accompanied by improvements in other glucometabolic factors like HbA1c and fasting insulin, as well as in the Framingham Risk Score. These results confirm our ‘Low-Insulin-Method’ concept for weight loss. Insulin stimulates lipogenesis and simultaneously inhibits lipolysis and storage of triglycerides which probably favours obesity [7]. The reduction in carbohydrate intake with or without formula diet might have contributed to these (beneficial) results.

Another positive effect of the multi-component SAMMAS intervention was the improvement in aspects (feeling of being hungry (*p* = 0.001) and eating control (*p* = 0.004; but not significant after Bonferroni correction)) in eating behaviour (ITT analysis). The intervention reduced feelings of hunger and indicated a trend for increased control regarding eating-associated actions, which have been shown to be disturbed in obese patients with or without type 2 diabetes [43]. In contrast, lifestyle-based intervention studies demonstrated meaningful improvements in eating behaviour after long-term mindful eating interventions in a small group of obese patients with type 2 diabetes [44] or without diabetes [45], which is in line with our findings.

There are strengths and limitations in the present trial that should be considered. First, the SAMMAS intervention study was highly accepted, thus, the overall dropout rate during 12 weeks of intervention and during the 52-weeks follow-up was 10% and 20%, respectively. This low dropout rate, especially after the intensive intervention phase, was also shown in another telemedical coaching study in patients with a metabolic syndrome [46]. The low dropout rate could be explained by the design of the study, with almost no barriers for the participants (e.g., 1:1 personal support, no additional costs). Most of the dropouts were due to individual reasons, including a change in employment. Second, employees of the Düsseldorf Catholic Hospital Group with overweight and obesity had been invited for participation in this study. Therefore, a selection bias by the investigation of only motivated employees cannot be excluded. However, the randomization procedure should have reduced any potential effect in the first 12 weeks of intervention. Furthermore, the baseline characteristics of both groups were not different. Another limitation of our study may be the lack of food records to screen for incorrect food compositions (e.g., the amount of carbohydrate in the diet, glycaemic index, fat or protein intake). On the other hand, we decided to abstain from food records, due to the well-known systematic errors associated with dietary records in an obese population [47]. However, we used coaching sessions to control and regulate food consumption. Furthermore, participants were encouraged to send pictures of their food and to note down their meals during the CGM phase, which should increase study compliance. Third, a potential limitation of the present study is the imputation of missing values due to the LOCF approach, which might have led to some reporting bias. The LOCF method is a conservative approach for longitudinal data to estimate treatment effects. Therefore, with this method, our results might have been underestimated. Fourth, as this RCT is a pilot study to examine the efficacy of SAMMAS, the number of participants per group was chosen to be in line with the guidelines of German health insurances for related patient training programs. Fifth, although there was no statistical difference between SI and WL regarding physical activity after 12 weeks of intervention, it should be considered that physical activity is a potential confounding factor which could have impacted all investigated results. As part of the seminar-based program (in theory and practice), participants were encouraged to increase physical activity (which was also supported and monitored by using pedometers), as well as to perform sport based on their own preference.

Furthermore, although a power calculation was performed to calculate an appropriate sample size to detect differences in weight change between both groups, the present small sample size could have had an impact on the power to detect differences in other parameters such as HbA1c. However, all statistical analyses were adjusted for baseline values, sex, age, and BMI.

The major strength of our study is the innovative, telemedical and multifactorial approach. Due to its low staff and economic requirements, our program could be easily implemented into occupational health care settings. Total costs for each participant in the present trial were around 700 EUR, including costs for equipment, coaching calls, salary for staff, and maintenance costs for the access to the online portal during the 52-week study period. When comparing the costs of our program with the health economic burden regarding obesity-related expenses (sick-leave days or drug costs) [48,49,50], SAMMAS could be a promising and long-term cost-effective treatment approach. Moreover, the possibility of repeating the program and the lack of side-effects underline the potential of our innovative lifestyle-based occupational health care program. In a follow-up project, we applied SAMMAS to a similar cohort from another company, which led to even better results after 52 weeks (e.g., weight reduction: −10 kg (95% CI [−13; −7]); Supplementary Figure S2). These findings illustrate the differences in generalisability between RCT and single-arm interventions and remarkably show the ambivalent problem of internal and external validity. Even RCT’s should be interpreted with caution, as selection bias (“understanding of informed consent”) or compliance problems in the control group can mask the potential effect of an intervention.

## 5. Conclusions

In conclusion, the Low-Insulin-Method included into the multi-component, occupational healthcare program SHAPE-AND-MOTION-Medical-Accompanied-Slimming (SAMMAS) could be an effective and promising new approach for the reduction in body weight and long-term weight loss maintenance in people with overweight and obesity. However, the present findings need to be confirmed in larger trials to increase generalizability.

## Figures and Tables

**Table 1:** Baseline characteristics.

	SI-Group (*n* = 15)	WL-Group (*n* = 15)	*p*
Anthropometrics			
Sex [*n*] (male%)	3 (20%)	2 (13%)	0.624
Age [years]	44 ± 9	49 ± 7	0.085
Weight [kg]	104 ± 25	92 ± 14	0.095
Body Mass Index [kg/m^2^]	35.1 ± 6.9	32.8 ± 6.1	0.346
Waist circumference [cm]	106 ± 19	101 ± 12	0.407
Fat mass [%]	43 ± 7	44 ± 8	0.486
SBP [mmHg]	121 ± 14	112 ± 31	0.354
DBP [mmHg]	69 ± 8	68 ± 7	0.636
Laboratory parameters			
Triglycerides [mg/dL]	180 ± 199	111 ± 48	0.208
Total cholesterol [mg/dL]	210 ± 46	214 ± 33	0.765
HDL cholesterol [mg/dL]	61 ± 16	62 ± 11	0.776
LDL cholesterol [mg/dL]	129 ± 38	140 ± 27	0.340
HbA1c [%] [mmol/mol]	6.2 ± 1.7 44 ± 19	5.5 ± 0.3 37 ± 3	0.167
Fasting blood glucose [mg/dL]	113 ± 41	95 ± 10	0.128
Fasting insulin [µU/mL]	11.8 ± 3.9	13.1 ± 7.9	0.576
HOMA-IR	3.4 ± 2.2	3.2 ± 2.2	0.928
Cardiometabolic risk			
FRS	7.6 ± 5.9	10.2 ± 5.2	0.250
Criteria of metabolic syndrome	2.1 ± 0.7	1.5 ± 1.0	0.540
Eating behaviour			
TFEQ—Cognitive control [au]	8.6 ± 3.0	8.9 ± 3.0	0.768
TFEQ—Suggestibility [au]	9.9 ± 3.6	8.6 ± 3.3	0.303
TFEQ—Hunger [au]	6.9 ± 3.2	6.1 ± 4.1	0.562
Quality of life			
SF12—Physical health [au]	48 ± 9	50 ± 9	0.385
SF12—Mental health [au]	35 ± 7	39 ± 5	0.131
Physical activity			
FFkA—Sports per week [min/week]	26 ± 47	113 ± 219	0.147
FFkA—Physically active per week [min/week]	338 ± 350	628 ± 941	0.276

Data are shown as mean ±SD or%. au, arbitrary unit; DBP, diastolic blood pressure; FFkA, Freiburger Questionnaire for Physical Activity; FRS, Framingham Risk Score; HDL, high-density lipoprotein; HOMA-IR, homeostasis model assessment of insulin resistance; LDL, low-density lipoprotein; HbA1c, haemoglobin A1c; SBP, systolic blood pressure; SF12, Short Form 12 Questionnaire; SI, starting intervention group; TFEQ, Three-factor Eating Questionnaire; WL, waiting list control group.

**Table 2:** Differences after 12 weeks of intervention.

	ETD	PP	PP	*p*	ETD	ITT	ITT	*p*
		SI-Group (*n* = 14)	WL-Group (*n* = 14)			SI-Group (*n* = 15)	WL-Group (*n* = 15)	
Anthropometrics								
Weight [kg]	−6.3 [−7.4; −4.5]	−7.8 [−9.7; −5.9]	−1.5 [−3.4; 0.5]	**<0.001**	−5.9 [−8.1; −4.1]	−7.3 [−9.3; −5.4]	−1.4 [−3.3; 0.6]	**0.001**
Weight [%]	−6.8 [−8.0; −3.9]	−8.4 [−12.2; −4.6]	−1.6 [−3.9; 0.8]	**<0.001**	−6.3 [−7.8; −3.7]	−8.0 [−11.5; −3.5]	−1.5 [−3.6; 0.6]	**<0.001**
BMI [kg/m^2^]	−2.3 [−3.4; −1.2]	−2.5 [−3.1; −1.9]	−0.5 [−1.1; 0.1]	**<0.001**	−2.1 [−3.1; −1.0]	−2.4 [−3.0; −1.8]	−0.4 [−1.1; 0.2]	**<0.001**
Waist circum-ference [cm]	−6.4 [−10.0; −2.8]	−7.7 [−10.1; −5.2]	−1.9 [−4.4; 0.5]	**<0.001**	−6.2 [−9.5; −2.3]	−7.6 [−9.8; −6.0]	−1.8 [−3.7; 0.1]	**<0.001**
Fat mass [%]	−2.7 [−4.3; −1.2]	−3.8 [−4.7; −2.8]	−0.3 [−1.3; 0.7]	**<0.001**	−2.2 [−3.1; −0.9]	−3.5 [−4.5; −2.5]	−0.3 [−1.3; 0.7]	**<0.001**
SBP [mmHg]	− 1.0 [−12; 10]	−11 [−22; −1]	−7 [−17; 6]	0.522	− 0.5 [−10; 8]	−10 [−20; −1]	−6 [−16; 3]	0.530
DBP [mmHg]	0 [−2;2]	−3 [−9; 4]	−3 [−9; 4]	1.000	−1 [−3;2]	−2 [−7; 3]	−3 [−8; 3]	0.990
Laboratory parameters								
Triglycerides [mg/dL]	−40 [−70; 10]	−62 [−134; 9]	−1 [−72; 72]	0.218	−25 [−55; 20]	−58 [−125; 9]	−1 [−67; 66]	0.220
Total cholesterol [mg/dL]	−6 [−32; 20]	−23 [−37; −9]	−11 [−25; 3]	0.200	−4 [−25; 16]	−21 [−35; −8]	−11 [−24; 3]	0.248
HDL cholesterol [mg/dL]	3 [−6; 9]	−3 [−8; 2]	−6 [−11; −1]	0.350	2 [−5; 7]	−3 [−7; 2]	−5 [−10; −1]	0.443
LDL cholesterol [mg/dL]	−9 [−14; 5]	−12 [−25; 2]	−2 [−15; 12]	0.161	−8 [−16; 4]	−11 [−24; 1]	−1 [−14; 11]	0.280
HbA1c [%] [mmol/mol]	−0.10 [−0.40; 0.30] −1.0 [−4.4; 3.3]	−0.57 [−1.08; −0.07] −6.2 [−11.8; −0.8]	−0.06 [−0.57; 0.44] −0.7 [−6.2; 4.8]	0.356	−0.08 [−0.32; 0.16] −0.9 [−3.5; 1.7]	−0.53 [−1.00; −0.06] −5.8 [−10.9; −0.7]	−0.06 [−0.53; 0.41] −0.7 [−5.3; 4.5]	0.167
Fasting blood glucose [mg/dL]	−3 [−16; 11]	−15 [−29; −1]	−1 [−15; 13]	0.104	−2 [−13; 9]	−13 [−26; −1]	−1 [−14; 12]	0.178
Fasting insulin [µU/mL]	−0.5 [−3.7; 2.7]	−3.5 [−5.9; −1.1]	−3.0 [−5.4; −0.6]	0.777	−0.3 [−3.0; 2.4]	−3.2 [−5.5; −1.0]	−2.8 [−5.1; −0.5]	0.780
HOMA-IR	−0.5 [−1.4; 0.7]	−1.5 [−2.4; −0.5]	−0.8 [−1.7; 0.2]	0.139	−0.4 [−1.2; 0.6]	−1.4 [−2.3; −0.4]	−0.7 [−1.6; 0.1]	0.217
Cardiometabolic risk								
Framingham Risk score	−0.4 [−1.0; 0.6]	−1.4 [−2.6; −0.2]	−0.8 [−2.0; 0.5]	0.152	−0.3 [−0.8; 0.5]	−1.3 [−2.1; −0.3]	−0.6 [−1.4; 0.2]	0.248
Criteria of metabolic syndrome	−0.2 [−0.6; 0.2]	−0.5 [−1.0; 0.1]	−0.1 [−0.6; 0.5]	0.277	−0.1 [−0.5; 0.4]	−0.4 [−0.9; 0.5]	0 [−0.4; 0.4]	0.420
Eating behaviour								
TFEQ—Cognitive control [au]	3.5 [2.7; 4.1]	5.7 [3.9; 7.5]	1.6 [−0.2; 3.4]	0.003	3.0 [2.1; 3.8]	5.3 [3.5; 7.1]	1.5 [−0.3; 3.3]	0.004
TFEQ—Suggestibility [au]	−2.2 [−3.0; −0.5]	−2.4 [−3.8; −0.9]	0.2 [−1.7; 1.2]	0.017	−1.6 [−2.4; −1.0]	−2.2 [−3.6; −0.8]	0.2 [−1.2; 1.6]	0.025
TFEQ—Hunger [au]	−1.7 [−2.5; −1.0]	−2.1 [−3.0; −1.3]	0.1 [−0.7; 1.0]	**0.001**	−1.2 [−1.9; −0.7]	−2.0 [−2.8; −1.2]	0.1 [−0.7; 0.9]	**0.001**
Quality of life								
SF12—Physical health [au]	0.4 [−1.0; 1.8]	1.6 [−1.6; 4.9]	0.9 [−2.4; 4.2]	0.748	0.2 [−0.7; 1.2]	1.5 [−1.5; 4.6]	0.9 [−2.2; 3.9]	0.755
SF12—Mental health [au]	−0.2 [−2.4; 2.1]	−0.1 [−4.2; 4.1]	0.2 [−4.0; 4.4]	0.928	−0.1 [−2.0; 1.9]	−0.1 [−4.2; 4.1]	−0.1 [−3.9; 3.8]	1.000
Physical activity								
FFkA—Sports per week [min/week]	40 [−60; 100]	61 [−16; 139]	−20 [−98; 57]	0.139	30 [−45; 85]	57 [−15; 129]	−19 [−91; 53]	0.147
FFkA—Physically active per week [min/week]	315 [−50; 452]	505 [193; 817]	79 [−233; 391]	0.058	225 [−70; 320]	471 [177; 765]	73 [−220; 367]	0.060

Data are shown as mean (95% CI). Bold *p*-values represent significance after Bonferroni correction (*p* = 0.00125). Multivariable regression analyses were carried out to investigate group differences (treatment effects) and were adjusted for age, sex, BMI, and baseline values. au, arbitrary unit; DBP, diastolic blood pressure; ETD, estimated treatment difference; FFkA, Freiburger Questionnaire for Physical Activity; HDL, high-density lipoprotein; HOMA-IR, homeostasis model assessment of insulin resistance; ITT, intention to treat analysis; LDL, low-density lipoprotein; HbA1c, haemoglobin A1c; PP, per protocol analysis; SF12, Short Form 12 Questionnaire; SBP, systolic blood pressure; TFEQ, Three-factor Eating Questionnaire.

## Supplementary Materials

The following are available online at https://www.mdpi.com/2072-6643/12/10/3004/s1, Figure S1: Detailed summary of all measurements, outcomes and time points for SI and WL, Figure S2: Mean changes in the present RCT compared to a confirmative single-arm trial, Table S1: Intra-group changes in the WL-group between waiting and intervention period.

## References

[1] Mattson M.P., Longo V.D., Harvie M. (2017). Impact of intermittent fasting on health and disease processes. Ageing Res. Rev..

[2] Afshin A., Forouzanfar M.H., Reitsma M.B., Sur P., Estep K., Lee A., Marczak L., Mokdad A.H., Moradi-Lakeh M., Naghavi M. (2017). Health Effects of Overweight and Obesity in 195 Countries over 25 Years. N. Engl. J. Med..

[3] O’Keefe S.J. (2019). The association between dietary fibre deficiency and high-income lifestyle-associated diseases: Burkitt’s hypothesis revisited. Lancet Gastroenterol. Hepatol..

[4] Reynolds A.N., Akerman A.P., Mann J. (2020). Dietary fibre and whole grains in diabetes management: Systematic review and meta-analyses. PLoS Med..

[5] Jacob S., Hauer B., Becker R., Artzner S., Grauer P., Löblein K., Nielsen M., Renn W., Rett K., Wahl H.G. (1999). Lipolysis in skeletal muscle is rapidly regulated by low physiological doses of insulin. Diabetologia.

[6] Meyer-Gerspach A.C., Cajacob L., Riva D., Herzog R., Drewe J., Beglinger C., Wölnerhanssen B.K. (2016). Mechanisms Regulating Insulin Response to Intragastric Glucose in Lean and Non-Diabetic Obese Subjects: A Randomized, Double-Blind, Parallel-Group Trial. PLoS ONE.

[7] Kolb H., Stumvoll M., Kramer W., Kempf K., Martin S. (2018). Insulin translates unfavourable lifestyle into obesity. BMC Med..

[8] Corkey B.E. (2012). Banting lecture 2011: Hyperinsulinemia: Cause or consequence?. Diabetes.

[9] Pories W.J., Dohm G.L. (2012). Diabetes: Have we got it all wrong? Hyperinsulinism as the culprit: Surgery provides the evidence. Diabetes Care.

[10] Kolb H., Kempf K., Röhling M., Martin S. (2020). Insulin: Too much of a good thing is bad. BMC Med..

[11] Davies M.J., D’Alessio D.A., Fradkin J., Kernan W.N., Mathieu C., Mingrone G., Rossing P., Tsapas A., Wexler D.J., Buse J.B. (2018). Management of hyperglycaemia in type 2 diabetes, 2018. A consensus report by the American Diabetes Association (ADA) and the European Association for the Study of Diabetes (EASD). Diabetologia.

[12] Davies M.J., D’Alessio D.A., Fradkin J., Kernan W.N., Mathieu C. (2018). Management of Hyperglycemia in Type 2 Diabetes, 2018. A Consensus Report by the American Diabetes Association (ADA) and the European Association for the Study of Diabetes (EASD). Diabetes Care.

[13] American Diabetes Association (2018). 4. Lifestyle Management: Standards of Medical Care in Diabetes-2018. Diabetes Care.

[14] Cecchini M., Sassi F., Lauer J.A., Lee Y.Y., Guajardo-Barron V., Chisholm D. (2010). Tackling of unhealthy diets, physical inactivity, and obesity: Health effects and cost-effectiveness. Lancet.

[15] Foster G.D., Wyatt H.R., Hill J.O., McGuckin B.G., Brill C., Mohammed B.S., Szapary P.O., Rader D.J., Edman J.S., Klein S. (2003). A randomized trial of a low-carbohydrate diet for obesity. N. Engl. J. Med..

[16] Astrup A., Meinert Larsen T., Harper A. (2004). Atkins and other low-carbohydrate diets: Hoax or an effective tool for weight loss?. Lancet.

[17] Esposito K., Maiorino M.I., Ciotola M., Di Palo C., Scognamiglio P., Gicchino M., Petrizzo M., Saccomanno F., Beneduce F., Ceriello A. (2009). Effects of a Mediterranean-style diet on the need for antihyperglycemic drug therapy in patients with newly diagnosed type 2 diabetes: A randomized trial. Ann. Intern. Med..

[18] Pitt C.E. (2016). Cutting through the Paleo hype: The evidence for the Palaeolithic diet. Aust. Fam. Physician.

[19] Walczyk T., Wick J.Y. (2017). The Ketogenic Diet: Making a Comeback. Consult. Pharm..

[20] Kempf K., Altpeter B., Berger J., Reuss O., Fuchs M., Schneider M., Gartner B., Niedermeier K., Martin S. (2017). Efficacy of the Telemedical Lifestyle intervention Program TeLiPro in Advanced Stages of Type 2 Diabetes: A Randomized Controlled Trial. Diabetes Care.

[21] Kempf K., Röhling M., Niedermeier K., Gärtner B., Martin S. (2018). Individualized Meal Replacement Therapy Improves Clinically Relevant Long-Term Glycemic Control in Poorly Controlled Type 2 Diabetes Patients. Nutrients.

[22] Röhling M., Kempf K., Banzer W., Berg A., Braumann K.M., Tan S., Halle M., McCarthy D., Pinget M., Predel H.G. (2020). Prediabetes Conversion to Normoglycemia Is Superior Adding a Low-Carbohydrate and Energy Deficit Formula Diet to Lifestyle Intervention-A 12-Month Subanalysis of the ACOORH Trial. Nutrients.

[23] Kempf K., Kruse J., Martin S. (2012). ROSSO-in-praxi follow-up: Long-term effects of self-monitoring of blood glucose on weight, hemoglobin A1c, and quality of life in patients with type 2 diabetes mellitus. Diabetes Technol. Ther..

[24] Kempf K., Kruse J., Martin S. (2010). ROSSO-in-praxi: A self-monitoring of blood glucose-structured 12-week lifestyle intervention significantly improves glucometabolic control of patients with type 2 diabetes mellitus. Diabetes Technol. Ther..

[25] Kempf K., Röhling M., Stichert M., Fischer G., Boschem E., Könner J., Martin S. (2018). Telemedical Coaching Improves Long-Term Weight Loss in Overweight Persons: A Randomized Controlled Trial. Int. J. Telemed. Appl..

[26] Kempf K., Röhling M., Martin S., Schneider M. (2019). Telemedical coaching for weight loss in overweight employees: A three-armed randomised controlled trial. BMJ Open.

[27] Kempf K., Dirk M., Kolb H., Hebestreit A., Bittner G., Martin S. (2012). The Da Vinci Medical-mental motivation program for supporting lifestyle changes in patients with type 2 diabetes. Dtsch Med. Wochenschr..

[28] Martin S., Kempf K. (2019). Das Neue Diabetes-Programm: Mit Protein-Shakes den Blutzucker Senken und Abnehmen (Deutsch) Taschenbuch.

[29] Fuller N.J., Jebb S.A., Laskey M.A., Coward W.A., Elia M. (1992). Four-component model for the assessment of body composition in humans: Comparison with alternative methods, and evaluation of the density and hydration of fat-free mass. Clin. Sci..

[30] Gallagher D., Heymsfield S.B., Heo M., Jebb S.A., Murgatroyd P.R., Sakamoto Y. (2000). Healthy percentage body fat ranges: An approach for developing guidelines based on body mass index. Am. J. Clin. Nutr..

[31] Ware J., Kosinski M., Keller S.D. (1996). A 12-Item Short-Form Health Survey: Construction of scales and preliminary tests of reliability and validity. Med. Care.

[32] Frey I., Berg A., Grathwohl D., Keul J. (1999). [Freiburg Questionnaire of physical activity--development, evaluation and application]. Soz Praventivmed..

[33] Stunkard A.J., Messick S. (1985). The three-factor eating questionnaire to measure dietary restraint, disinhibition and hunger. J. Psychosom. Res..

[34] D’Agostino R.B., Vasan R.S., Pencina M.J., Wolf P.A., Cobain M., Massaro J.M., Kannel W.B. (2008). General cardiovascular risk profile for use in primary care: The Framingham Heart Study. Circulation.

[35] Lindstrom J., Louheranta A., Mannelin M., Rastas M., Salminen V., Eriksson J., Uusitupa M., Tuomilehto J. (2003). The Finnish Diabetes Prevention Study (DPS): Lifestyle intervention and 3-year results on diet and physical activity. Diabetes Care.

[36] Knowler W.C., Barrett-Connor E., Fowler S.E., Hamman R.F., Lachin J.M., Walker E.A., Nathan D.M. (2002). Reduction in the incidence of type 2 diabetes with lifestyle intervention or metformin. N. Engl. J. Med..

[37] Kosaka K., Noda M., Kuzuya T. (2005). Prevention of type 2 diabetes by lifestyle intervention: A Japanese trial in IGT males. Diabetes Res. Clin. Pract..

[38] Leslie W.S., Taylor R., Harris L., Lean M.E. (2017). Weight losses with low-energy formula diets in obese patients with and without type 2 diabetes: Systematic review and meta-analysis. Int. J. Obes..

[39] Kahleova H., Belinova L., Malinska H., Oliyarnyk O., Trnovska J., Skop V., Kazdova L., Dezortova M., Hajek M., Tura A. (2014). Eating two larger meals a day (breakfast and lunch) is more effective than six smaller meals in a reduced-energy regimen for patients with type 2 diabetes: A randomised crossover study. Diabetologia.

[40] Jackness C., Karmally W., Febres G., Conwell I.M., Ahmed L., Bessler M., McMahon D.J., Korner J. (2013). Very low-calorie diet mimics the early beneficial effect of Roux-en-Y gastric bypass on insulin sensitivity and beta-cell Function in type 2 diabetic patients. Diabetes.

[41] Appel L.J., Clark J.M., Yeh H.C., Wang N.Y., Coughlin J.W., Daumit G., Miller E.R., Dalcin A., Jerome G.J., Geller S. (2011). Comparative effectiveness of weight-loss interventions in clinical practice. N. Engl. J. Med..

[42] Holzapfel C., Merl M., Stecher L., Hauner H. (2016). One-Year Weight Loss with a Telephone-Based Lifestyle Program. Obes. Facts.

[43] Loffler A., Luck T., Then F.S., Sikorski C., Kovacs P., Bottcher Y., Breitfeld J., Tonjes A., Horstmann A., Loffler M. (2015). Eating Behaviour in the General Population: An Analysis of the Factor Structure of the German Version of the Three-Factor-Eating-Questionnaire (TFEQ) and Its Association with the Body Mass Index. PLoS ONE.

[44] Miller C.K., Kristeller J.L., Headings A., Nagaraja H. (2014). Comparison of a mindful eating intervention to a diabetes self-management intervention among adults with type 2 diabetes: A randomized controlled trial. Health Educ. Behav..

[45] Nurkkala M., Kaikkonen K., Vanhala M.L., Karhunen L., Keranen A.M., Korpelainen R. (2015). Lifestyle intervention has a beneficial effect on eating behavior and long-term weight loss in obese adults. Eat. Behav..

[46] Luley C., Blaik A., Gotz A., Kicherer F., Kropf S., Isermann B., Stumm G., Westphal S. (2014). Weight loss by telemonitoring of nutrition and physical activity in patients with metabolic syndrome for 1 year. J. Am. Coll. Nutr..

[47] Bhanpuri N.H., Hallberg S.J., Williams P.T., McKenzie A.L., Ballard K.D., Campbell W.W., McCarter J.P., Phinney S.D., Volek J.S. (2018). Cardiovascular disease risk factor responses to a type 2 diabetes care model including nutritional ketosis induced by sustained carbohydrate restriction at 1 year: An open label, non-randomized, controlled study. Cardiovasc. Diabetol..

[48] Lehnert T., Stuhldreher N., Streltchenia P., Riedel-Heller S.G., Konig H.H. (2014). Sick leave days and costs associated with overweight and obesity in Germany. J. Occup. Environ. Med..

[49] Tremmel M., Gerdtham U.G., Nilsson P.M., Saha S. (2017). Economic Burden of Obesity: A Systematic Literature Review. Int. J. Environ. Res. Public Health.

[50] Arterburn D.E., Maciejewski M.L., Tsevat J. (2005). Impact of morbid obesity on medical expenditures in adults. Int. J. Obes..

