# Supplementary material for: Weight Reduction by the Low-Insulin-Method—A Randomized Controlled Trial
Source: Nutrients. 2020 Sep 30;12(10):3004. doi: 10.3390/nu12103004 (PMC7601801; doi:10.3390/nu12103004)
Supplement: Supplementary file 1 [file nutrients-12-03004-s001.pdf]

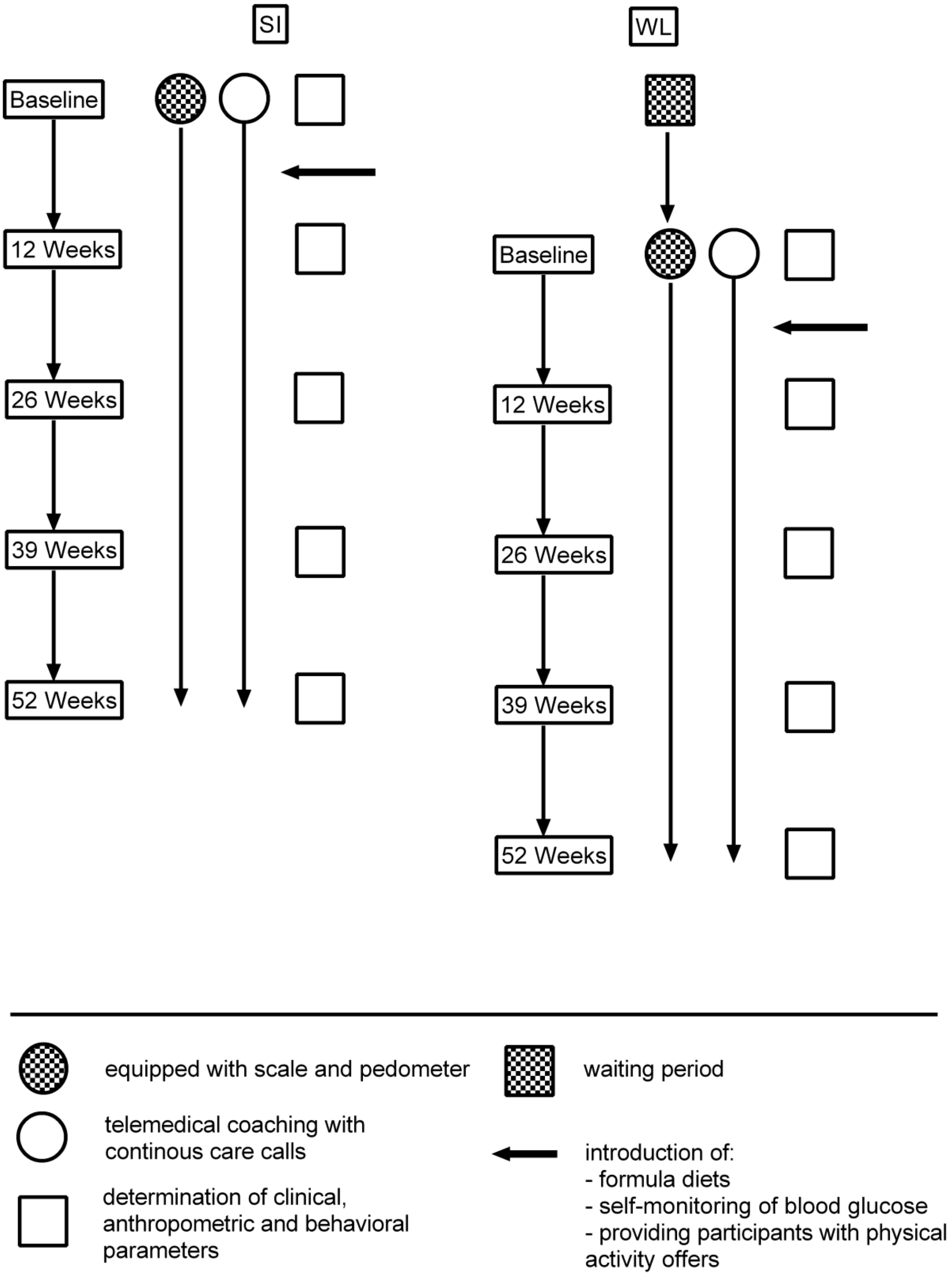
SI
WL
Baseline
12 Weeks
26 Weeks
39 Weeks
52 Weeks
Baseline
12 Weeks
26 Weeks
39 Weeks
52 Weeks
equipped with scale and pedometer
waiting period
telemedical coaching with
continous care calls
introduction of:
- formula diets
- self-monitoring of blood glucose
- providing participants with physical
activity offers
determination of clinical,
anthropometric and behavioral
parameters
